# Cellular profiling identifies targetable T cell phenotypes in lymphocytic variant hypereosinophilic syndrome

**DOI:** 10.1172/JCI190853

**Published:** 2025-11-03

**Authors:** Kristy Tefft, Amy Wang, Zachary Z. Reinstein, Yue Zhang, Arundhati Pillai, Sunghee Hwang, Spencer Ng, Raymond J. Cho, Jeffrey B. Cheng, Fei Li Kuang, Brett King, Jaehyuk Choi

**Affiliations:** 1 Department of Dermatology, Northwestern University, Chicago, Illinois, USA.; 2 Department of Dermatology, Indiana University School of Medicine, Indianapolis, Indiana, USA.; 3 Department of Dermatology, UCSF, San Francisco, California, USA.; 4 Dermatology Service, San Francisco Veterans Administration Health Care System, San Francisco, California, USA.; 5 Department of Allergy and Immunology, Northwestern University, Chicago, Illinois, USA.; 6 Dermatology Physicians of Connecticut, Fairfield, Connecticut, USA.; 7 Department of Dermatology, University of Texas Southwestern, Dallas, Texas, USA.

**Keywords:** Dermatology, Immunology, Lymphomas, Molecular biology, T cells

## Abstract

With cell-by-cell mapping, we charted immune cells in lymphocytic-variant hypereosinophilic syndrome, found drug targets, and showed targeted therapy removed disease-causing cells and eased symptoms.

**To the Editor:** In lymphocytic variant hypereosinophilic syndrome (LHES), an aberrant Th2-skewed lymphocyte clone (commonly CD3^–^CD4^+^) drives tissue eosinophilia (1). To elucidate mechanisms, we performed multimodal single-cell RNA-seq (scRNA-seq) (2) on peripheral blood mononuclear cells from four patients with LHES (patients 1–4) and one with idiopathic hypereosinophilic syndrome (iHES) (patient 5).

Patients 1–4 met LHES criteria, two of whom reported inflammatory arthritis (Supplemental Table 1 and Figure 1A; supplemental material available online with this article; https://doi.org/10.1172/JCI190853DS1). Flow cytometry identified a circulating CD3^–^CD4^+^ T cell population in patients with LHES. Serum IL-5 was elevated in the 2 patients with LHES tested but normal in the patient with iHES. All patients received JAK inhibitors, which partially improved symptoms/eosinophilia. Two refractory patients received additional IL-5 blockade (Supplemental Table 1).

scRNA-seq identified two clonally expanded CD3^–^CD4^+^ clusters only in patients with LHES (Figure 1B and Supplemental Figure 1A). Clusters 1 and 2 (predominantly patient 4 or 3, respectively) expressed markers of central memory (CD45RA^–^CD45RO^+^CD127^+^CD62L^+^) and effector memory T cells(CD45RA^–^CD45RO^+^CD127^+^CD62L^–^), respectively (Supplemental Figure 1, A and B). Both expressed activation (CD226, ICOS) but not exhaustion markers (PD-1, CD39); cluster 2 showed higher expression of additional activation markers (CD38, HLA-DR, CD69, CD25) (Supplemental Figure 1A). CD3^–^CD4^+^ cells from these patients are predominantly one phenotype or cluster, regardless of timepoint (Supplemental Figure 1B). TCR sequencing demonstrated a productive monoclonal αβ-TCR gene rearrangement present within CD3^–^CD4^+^ clusters in all LHES patients, confirming they are bona fide αβ-T cells despite no surface CD3 (Figure 1C).

CD3^–^CD4^+^ cells had upregulation of canonical Th2-associated transcription factors (*GATA3*, *BATF*), cytokine (*IL13*), and surface proteins (*PTGDR2*, *CCR8*, *CCR4*) (likelihood ratio test; false discovery rate (FDR) < 0.01) (Supplemental Figure 1C). CD3^–^CD4^+^ clusters also expressed cytokines not unique to Th2 cells, including *OSM*, *CSF2*.

Next, we performed single-cell regulatory network inference and clustering (SCENIC), which predicted upregulated activity of Th2-associated transcription factor regulons (*GATA3*, *BATF*, *MAF*) (Supplemental Figure 1D), suggesting CD3^–^CD4^+^ lymphocytes share transcriptional networks with CD3^+^CD4^+^ Th2 cells (3). Therefore, we compared LHES cells to Th2 cells from allergic disease patients (3). They coclustered with Th2 memory-like cells. Nonetheless, they had higher expression of activator protein-1 (AP-1) complex members (*JUN*, *FOS*), receptors (*CCR4*, *CD52*), and cytokines (*TNF*, *TGFB1*) (Wilcoxon rank sum test, *P*_adj_ < 0.05) (Figure 1D). CCR4 and CD52 are targetable by FDA-approved mogamulizumab and alemtuzumab, respectively. TNFα (*TNF*) is a targetable Th1-associated cytokine that may contribute to arthritic symptoms in LHES (Figure 1D and Supplemental Table 2).

Additional SCENIC analysis predicted increased regulon activity of AP-1 members (*JUN*, *JUNB*, *FOS*) in CD3^–^CD4^+^ cells. AP-1 members mediate a variety of cellular processes in T cells, including persistence and proliferation (4). They also upregulated other regulon activity associated with self-renewal/stemness in T cells (*TCF7*) and other cell types (*HMGA1*, *SPI1*) (Figure 1E and Supplemental Figure 2) (3).

We next examined expression of CD3 transcripts. Unlike previous studies (5), we found decreased expression of only one CD3 component, CD3ζ, similarly found in lupus (6) (FDR < 0.0001) (Supplemental Figures 1, C and E). siRNA knockdown of CD3ζ confirmed that decreased expression is sufficient to induce loss of surface CD3/TCR (Supplemental Figure 1F).

Without surface CD3, the driver of CD3^–^CD4^+^ T cell proliferation was unclear. CellChat analysis predicted autocrine IL-7 signaling among CD3^–^CD4^+^ cells (*P* < 0.05) (Figure 1F). This has been shown to be sufficient to support antigen-independent proliferation (7). IL-7 inhibition may thus represent a therapeutic target in patients with LHES (Supplemental Table 2).

Patient 4, with high CCR4 expression, having exhausted conventional treatment options with rapid clinical deterioration, received mogamulizumab, as recently described (8). Cutaneous and joint symptoms resolved by 3 months. Flow cytometry at 6 weeks did not detect CD3^–^CD4^+^ T cells, suggesting successful depletion of the LHES clone (Figure 1G).

Lastly, we identified DNA variants (gain-of-function CTNNB1 p.S45P) in patient 3’s CD3^–^CD4^+^ T cells (Figure 1H). During disease progression on JAK inhibitor, the clone acquired a STAT3 p.G618R variant (previously identified in an untreated LHES case) (9), which was absent pretreatment (Figure 1H), suggesting a potential mechanism of acquired resistance to JAK inhibitors.

In summary, scRNA-seq identifies distinctive molecular features of LHES biology and potential therapeutic vulnerabilities and resistance mechanisms.

## Funding support

This work is the result of NIH funding, in whole or in part, and is subject to the NIH Public Access Policy. Through acceptance of this federal funding, the NIH has been given a right to make the work publicly available in PubMed Central.

- National Cancer Institute grant F30CA278298 (ZZR).
- NIH grant 1DP2AI136599-01 (JBC).
- Merit Review Award from the Veterans Health Administration Office of Research and Development (I01CX002608) (JBC).
- The LEO Foundation (LF-OC-22-001029) (JBC).

## Supplementary Material

Supplemental data

Supporting data values

## Figures and Tables

**Figure 1:**
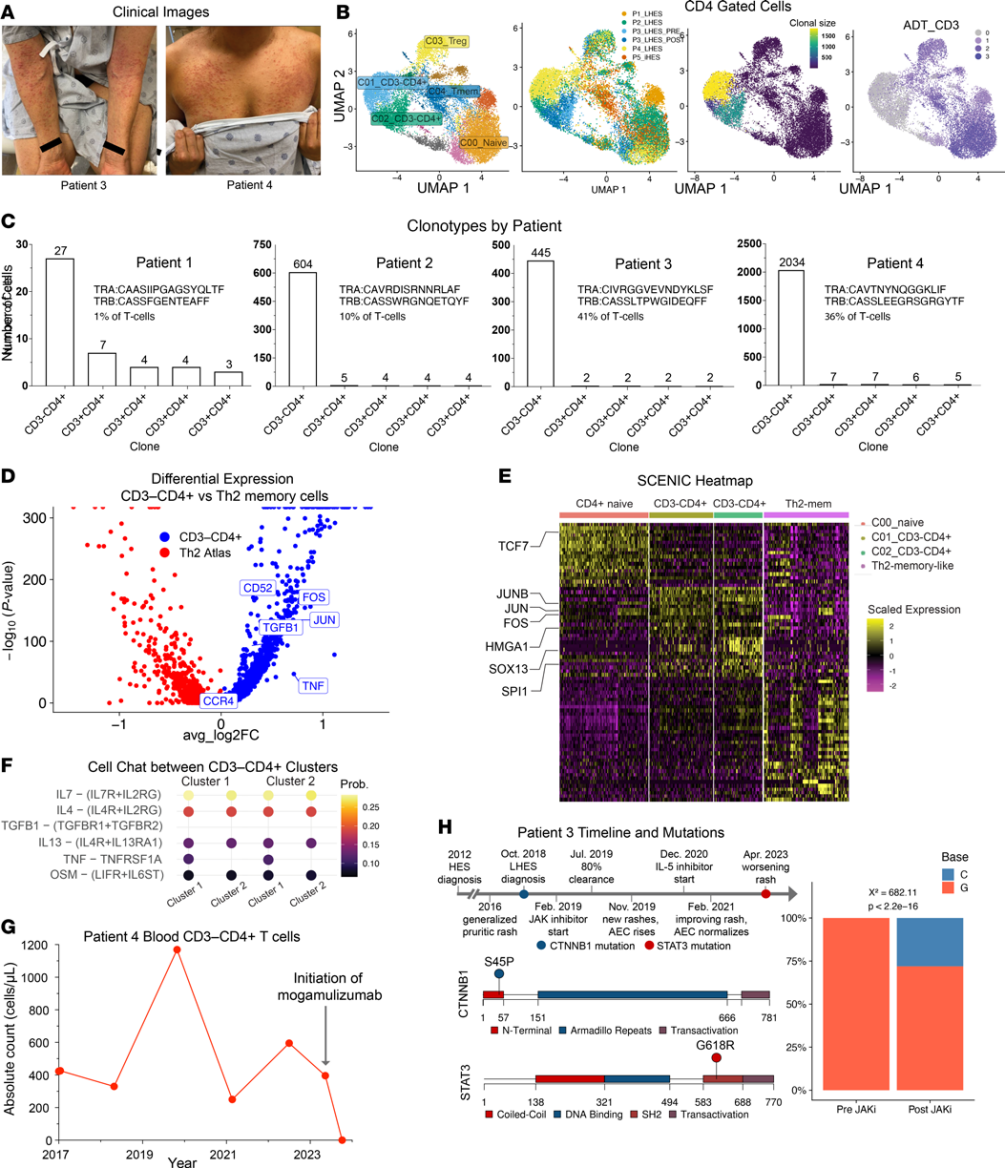
CD3^–^CD4^+^ lymphocytes are unique Th2-like cells and express targetable surface markers and cytokines. (**A**) Images of patients 3 and 4. (**B**) UMAPs of CD4-gated cells aggregated from all samples (*n* = 6) grouped/colored as indicated. Reg/mem, regulatory/memory. (**C**) Top 5 clonotypes per sample by cell number. (**D**) Volcano plot of differentially expressed genes between LHES-associated CD3^–^CD4^+^ versus atlas Th2 memory-like cells (*P*_adj_ < 0.05). (**E**) Heatmap of SCENIC regulons of naive CD4^+^ cells and CD3^–^CD4^+^ clusters from patients with LHES, atlas Th2 memory-like cells. (**F**) CellChat analysis among CD3^–^CD4^+^ T cells. (**G**) Absolute blood CD3^–^CD4^+^ T cell count in patient 4. (**H**) Patient 3 clinical course timeline; CTNNB1/STAT3 variant maps. Bar chart of posttreatment variant prevalence confirmed with amplicon DNA sequencing.

## Version 1 11/03/2025

Electronic publication
