# Supplementary material for: Cellular profiling identifies targetable T cell phenotypes in lymphocytic variant hypereosinophilic syndrome
Source: J Clin Invest. 2025 Nov 3;135(21):e190853. doi: 10.1172/JCI190853 (PMC12578380; doi:10.1172/JCI190853)
Supplement: Supplemental data [file jci-135-190853-s268.pdf]

Supplementary Figures and Methods to:
Article type: Clinical Medicine Research Letter
Title: Cellular profiling identifies targetable T-cell phenotypes in lymphocytic variant hypereosinophilic syndrome
Authors: Kristy Tefft,[1*] Amy Wang,[1*] Zachary Z. Reinstein,[1*] Yue Zhang,[2] Arundhati Pillai,[1] Sunghee Hwang,[1] Spencer Ng,[3] Raymond J. Cho,[4] Jeffrey Cheng,[4] Fei Li Kuang,[5] Brett King,[6] Jaehyuk Choi[1,7]
[1]Department of Dermatology, Northwestern University Feinberg School of Medicine, Chicago, IL, USA.
[2]Department of Dermatology, Indiana University School of Medicine, Indianapolis, IN, USA.
[3]Division of Dermatology, Department of Medicine, and Department of Pathology and Immunology, Washington University School of Medicine, St. Louis, MO, USA.
[4]Department of Dermatology, University of California, San Francisco, CA, USA.
[5]Division of Allergy and Immunology, Department of Medicine, Northwestern University Feinberg School of Medicine, Chicago, IL, USA.
[6]Dermatology Physicians of Connecticut, Fairfield, CT, USA.
[7]Center for Cellular Therapies and Cancer Immunology, University of Texas Southwestern, Dallas, TX, USA
*K.T., A.W., and Z.R. contributed equally to this study.
Corresponding author:
Jaehyuk Choi
Robert H. Lurie Comprehensive Cancer Center
Northwestern University
303 E. Superior St, Suite 5-115
Chicago, IL 60611
Tel: 312-503-4108
Email: jaehyuk.choi@northwestern.edu
Word count: 1187
References: 11
Figures: 1
Supplemental Figures: 2
Supplemental Tables: 2
Key words: lymphomas; molecular biology; T-cells
Acknowledgements: Funding for this study was provided by the Bakewell Foundation (J. Choi) and the Leukemia and Lymphoma Society grant 1377-21 (J. Choi).
Conflicts of interests: **J. Choi** is a stakeholder and cofounder of Moonlight Bio, which is unrelated to these studies. **B. King** has served on advisory boards and/or is a consultant and/or is a clinical trial investigator and/or is on a Data Monitoring Committee for Abbvie, AltruBio Inc, Almirall, Amgen, AnaptysBio, Apogee Therapeutics, Aquestive, Arena Pharmaceuticals, Aslan Pharmaceuticals, Bristol Meyers Squibb, Concert Pharmaceuticals Inc, Equillium, GSK, Horizon Therapeutics, Eli Lilly and Company, Incyte Corp, Janssen Pharmaceuticals, LEO Pharma, Merck, Otsuka/Visterra Inc, Pfizer Inc, Q32 Bio Inc, Regeneron, Sanofi Genzyme, Sun

Pharmaceutical, Takeda, TWi Biotechnology Inc, and Ventyx Biosciences Inc. He has served on speaker bureaus for Abbvie, Incyte, Eli Lilly, Pfizer, Regeneron and Sanofi Genzyme. He is a scientific advisor for BiologicsMD. **F.L. Kuang** receives research funding from AstraZeneca and speaker fees from both AstraZeneca and GlaxoSmithKline. **Z. Reinstein** has received consultant fees from OpenAI. **R.J. Cho** receives research support from Bristol-Myers Squibb, Regeneron, Janssen, and Sun Pharmaceuticals. **J. Cheng** has been an investigator for Sun Pharmaceutical Industries, LEO Pharmaceuticals, Sanofi, and Regeneron (grants to their institution). **K. Tefft, A. Wang, Y. Zhang, A. Pillai, S. Hwang,** and **S. Ng** have no conflicts of interest. The authors declare that none of these financial disclosures affected the content presented in the manuscript.

Statement on prior presentation: Presented in mini-symposium format at the 2024 Annual Meeting of the Society for Investigational Dermatology, Dallas, TX, May 18, 2024.

Patient consent: Patient consent is available on request.

Supplemental Table 1. Clinical Characterization of Lymphocytic Variant Hypereosinophilic Syndrome

	HES_1	HES_2	HES_3	HES_4	HES_5
**Age at diagnosis, gender**	27, male	58, female	60, female	31, female	74, female
**HES type**	L-HES	L-HES	L-HES	L-HES	iHES
**Blood eosinophil count (cells/mm^3) prior to JAKi**[1]	2101	2,819	12,770	6,450	3,067
**Serum IL-5 (pg/mL)**[2]	n/a	9	n/a	170.9	<5
**Serum IgE (IU/mL)**[2]	824	>5,000	30,463	1,342	1,009
**Flow cytometric analysis of peripheral blood**	abnormal T cell population CD3- CD4+ CD7- (<0.6% of total cellular events)	abnormal T cell population CD3- CD4+ CD5++ CD22++ CD7dim (3-5% of total cellular events)	abnormal T cell population CD3- CD4+ CD5+ CD2+ CD7- (5.6% of total cellular events)	abnormal T cell population CD3- CD4+ CD5++ CD8- CD2+ CD7 variable (3% of total cellular events)	A definitively abnormal T-cell population not identified
**Comorbidities**	unknown	unknown	rheumatoid arthritis, schizoaffective disorder, asthma, hypothyroidism	rheumatoid arthritis	diabetes mellitus, hypertension
**Prior treatments**	prednisone	prednisone, mycophenolate mofetil, extracorporeal photopheresis	prednisone, hydroxyzine	prednisone, omalizumab, dupilumab	prednisone, omalizumab, imatinib, azathioprine
**Follow-up period taking JAKi, months**	6	3	54	66	63
**Blood eosinophil count (cells/mm^3) on JAKi monotherapy**[1,3]	545	771*	6,415	4,166	930
**Blood eosinophil count (cells/mm^3) on JAK + IL-5 inhibition**[4]	n/a	n/a	1,143	2,472	n/a
**Symptoms pre-JAKi**	severe pruritus, erythematous edematous papules and	moderate pruritus, eczematous and lichenified plaques	severe pruritus, 70% BSA erythematous papules coalescing into	severe pruritus, erythematous papules on trunk and arms	severe pruritus, erythematous papules and

	plaques on extremities	on the head, neck, and extremities	plaques on neck, trunk, and extremities		plaques on extremities
**Symptoms post-JAKi**	no pruritus, clear skin	mild pruritus, mild eczematous dermatitis	moderate pruritus, 25% BSA erythematous papules; intermittent severe flares	moderate pruritus, erythematous papules coalescing into plaques; intermittent severe flares	no pruritus, clear skin

[1]Blood eosinophil count is the average of 2 or more values measured at least 1 month apart.
[2]Peak values measured prior to initiation of JAK inhibitor except for patient 4 who was treated with upadacitinib and mepolizumab during IL-5 measurement.
[3]Values are for tofacitinib, ruxolitinib, or upadacitinib monotherapy.
[4]Values are for combination therapy with JAK inhibitor and mepolizumab or benralizumab.
*Patient 2 was also taking low dose (5-10mg) prednisone throughout duration of tofacitinib treatment.

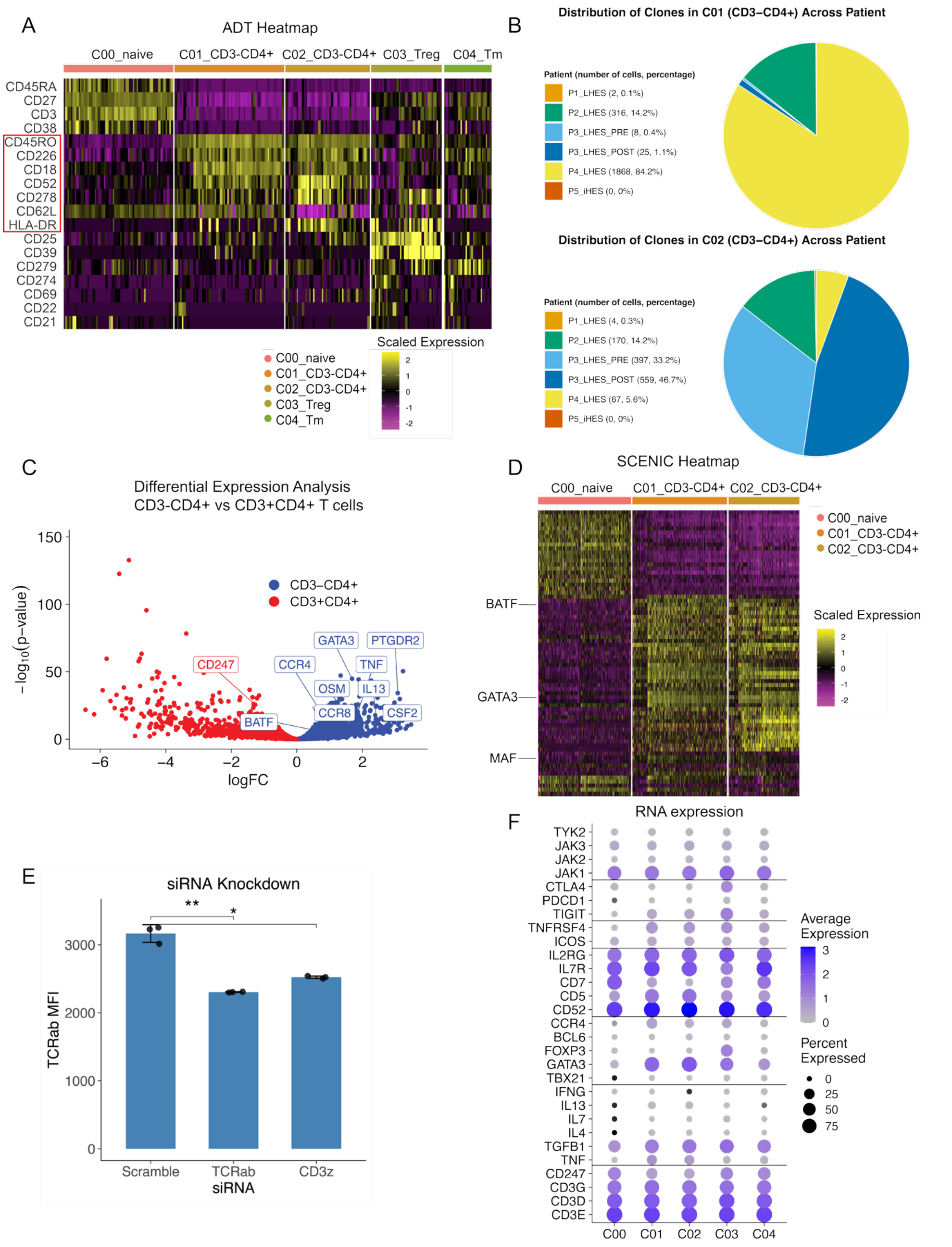

Supplemental Figure 1. Atypical Lymphocytes are Central or Effector Memory Th2-like cells. (A) Heatmap of surface marker expression using antibody-derived tags (ADTs) of five

largest CD4+ clusters of cells aggregated from all patient samples, including naïve T-cells (C00_naive), atypical LHES clusters (C01_CD3-CD4+, C02_CD3-CD4+), T regulatory cells (C03_Treg), and T memory cells (C04_Tmem). Red box highlights surface proteins expressed by CD3-CD4+ clusters. (B) Pie chart showing the distribution of expanded CD4+ clones ($n>2$) by patient sample between CD3-CD4+ cluster 1 and cluster 2. (C) Volcano plot of pseudobulk analysis showing differentially expressed genes of interest in atypical CD3-CD4+ population vs CD3+CD4+ cells (LRT; $FDR<0.005$). CD247, CD3ζ. (D) Heatmap of Single-cell regulatory network inference and clustering (SCENIC) regulon expression of atypical CD3-CD4+ clusters (C01_CD3-CD4+, C02_ CD3-CD4+). Th2-associated transcription factors are labeled. (E) Average RNA expression dot plot of largest CD4+ clusters, including naïve T-cells (C00_naive), atypical LHES clusters (C01_CD3) CD4+, C02_CD3-CD4+), T regulatory cells (C03_Treg), and T memory cells (C04_Tmem). (F) Jurkat CD4 T-cells were electroporated with siRNAs targeting scramble control, TCRa/b, and CD3z. The median fluorescence index (MFI) of TCRa/b was significantly decreased in both the TCRa/b, and CD3z conditions (Student t-test two-sided, $p= 0.012$).

Supplemental Table 2. Potential Therapeutic Targets in Lymphocytic Variant Hypereosinophilic Syndrome

**surface molecules**	**drug name**	**drug class**	**phase**
CCR4	mogamulizumab	depleting monoclonal antibody	FDA approved
CD52	alemtuzumab	depleting monoclonal antibody	FDA approved
CRTH2	timapiprant, multiple	competitive CRTH2 antagonists	clinical trials
CD5	MT-101	anti-CD5 CAR T-cells	clinical trials
CCR8	RO7502175	depleting monoclonal antibody	clinical trials
**cytokines**			
IL-5	mepolizumab	anti-IL-5 monoclonal antibody	FDA approved
TNFα	adalimumab<br>infliximab<br>etanercept<br>certolizumab pegol<br>golimumab	monoclonal antibodies,<br>fusion protein (etanercept)	FDA approved
IL-7	A7R-ADC, B12, 4A10, 2B8	antibody-drug conjugate (A7R-ADC),<br>neutralizing monoclonal antibody (B12),<br>depleting antibodies	clinical, preclinical trials
OSM	multiple	anti-OSM monoclonal antibodies,<br>small molecule inhibitors	clinical, preclinical trials
GM-CSF	lenzilumab	anti-GM-CSF monoclonal antibody	clinical trials

Surface molecules and cytokines expressed by CD3-CD4+ aberrant lymphocytes that offer potential therapeutic targets in various stages of investigation.

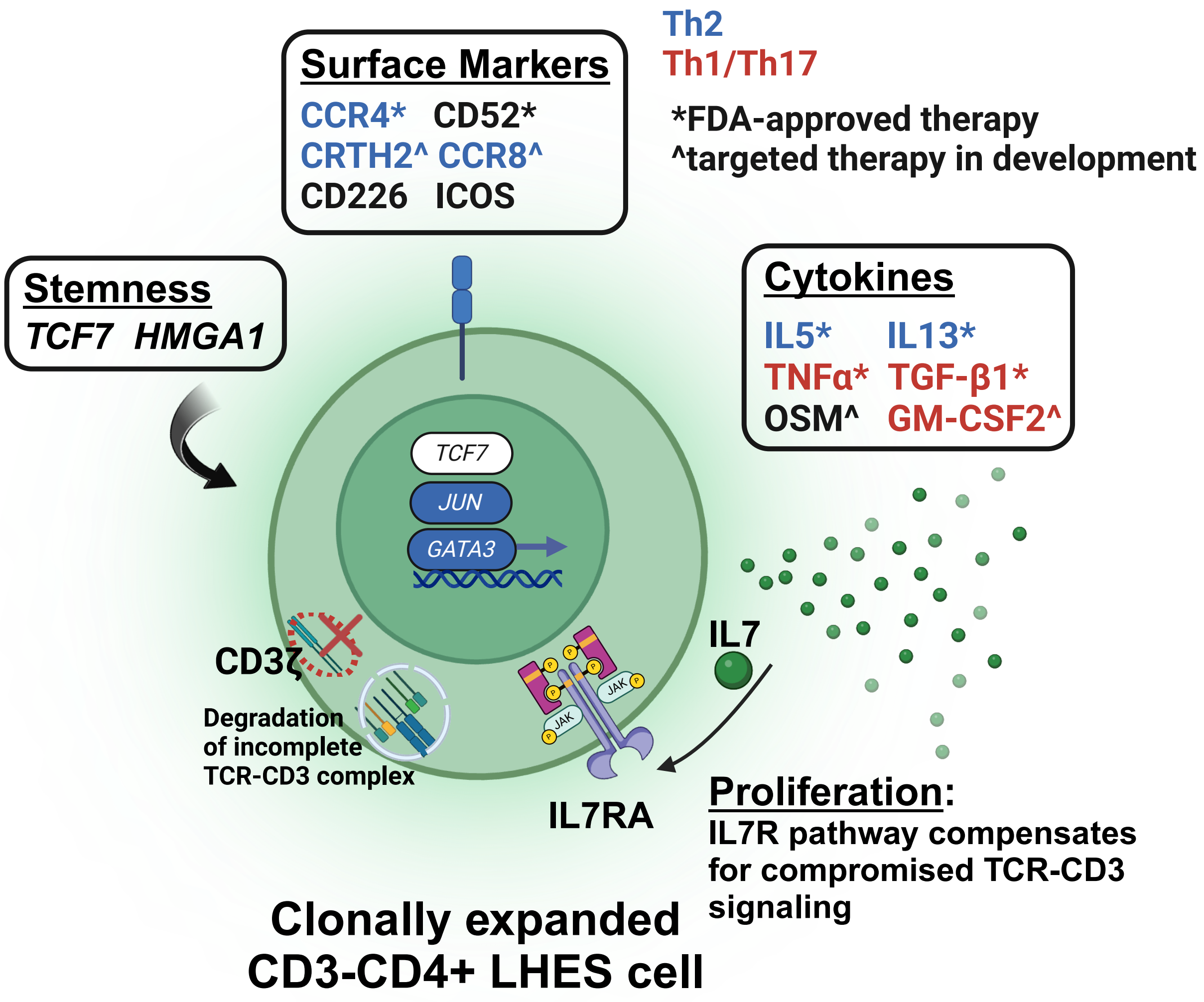

Supplemental Figure 2. Schematic model of CD3-CD4+ Lymphocyte. Diagram illustrating important surface markers, cytokines, and transcription factors. Therapeutic targets in various stages of investigation are highlighted. Proposed mechanism of CD3 loss via CD3 downregulation and IL-7 driven proliferation in the absence of surface TCR complex. Created in BioRender. Choi, J. (2025) https://biorender.com/r28f431

SUPPLEMENTARY METHODS

Sex as a Biological Variable.

Our study included 4 patients with LHES of whom 3 were female and 1 male. Results were similar for both sexes. The singular case of idiopathic HES was female.

Inclusion Criteria and Clinical Classification

4 patients with a confirmed diagnosis of LHES were included in this study. 1 patient was diagnosed with idiopathic HES. The diagnosis of LHES was made for all cases after meeting the following criteria(1):

- AEC >1.5×10^9/L for more than 6 months
- Evidence of tissue damage (all patients had skin lesions demonstrated on biopsy to have infiltrating eosinophils)
- Presence of a circulating abnormal T-cell population as demonstrated by flow cytometry immunophenotyping
- All other primary and secondary causes of HES were excluded

The patient with iHES met all of the above criteria but no abnormal T-cell population was detected, therefore rendering a diagnosis of idiopathic HES.

LHES and iHES specimens used for molecular studies were obtained from the PBMCs of patients after informed consent was obtained. Two LHES patients were published previously(2, 3).

Single Cell Isolation and Sequencing

Cryopreserved peripheral blood mononuclear cells (PBMCs) were thawed, labeled, and prepared for 10x Genomics droplet encapsulation following a modified protocol adapted from the 10x Genomics and BioLegend TotalSeq C protocols. Cells were blocked using Human TruStain FcX™ Fc blocking reagent (BioLegend, No.422302) and stained with TotalSeq™ C (BioLegend, Supporting Data: “ADT Antibodies”) and hashing antibodies (BioLegend; No.394661, No.394663, No.393665, No.394667, No.394669). Live cells were sorted using fluorescence-activated cell sorting (FACS) based on negative selection of ApoTracker Green (BioLegend) fluorescence, a marker for apoptotic cells. The cells were slow-frozen at -80°C before being transferred to liquid nitrogen for long-term storage. Thawing and final sample preparation for encapsulation were performed immediately before loading onto the 10x 5’ v2 Chromium platform. Sequencing parameters were set for the 5' cDNA, αβ VDJ, and ADT libraries according to the targeted cell number for single-cell RNA sequencing. αβ TCR libraries were prepared following the 10x Genomics Single Cell V(D)J protocol (4).

scRNA-seq Pre-Processing and Clustering

FASTQ files containing gene expression and feature barcodes (ADT) were aligned to the human GRCh38.p13 primary assembly genome (Ensembl Release 98, GENCODE v32) using CellRanger v6.1.0. Ambient RNA was removed using the CellBender (5) remove-background function with the default parameters of 150 epochs and 0.01 FPR. Processed read counts for each sample were imported as Seurat objects. We then filtered the data based on mitochondrial reads (25% cut-off) and minimum RNA genes (200 genes). Doublets were removed using DoubletFinder (6) with the default pN of 0.25 and the optimal pK value calculated with the "find.pK" function. Cells were annotated with ProjectTILS (7) and SingleR using Monaco and Human Primary Cell Atlas (HPCA) reference datasets. Data were merged into a single Seurat object. We performed standard Seurat (v4.3.0) weighted nearest neighbors clustering, Harmony (v1.2.0) batch correction, and manual cell cluster annotation based on marker genes, including transcription factors, effector molecules, and previously annotated cell types. Clusters annotated as CD4 cells were further sub-clustered. Contaminating cells were filtered based on marker gene expression. Differential gene expression was performed using the default Seurat pipeline.

Single cell TCR-seq Processing

FASTQ files for 5′ αβ V(D)J libraries were processed with Cell Ranger v3.0.2 (cellranger vdj, 10x Genomics) using the prebuilt human V(D)J reference refdata-cellranger-vdj-GRCh38-alts-ensembl-4.0.0. Using default cellranger vdj parameters, high-confidence, cell-associated contigs were retained, and productive rearrangements were defined per Cell Ranger/IMGT criteria (in-frame TRA/TRB transcripts spanning a valid CDR3 with no premature stop codon). For each cell barcode, productive TRA and TRB chains were paired to define αβ clonotypes by the exact CDR3α+CDR3β amino-acid sequences (with TRAV/TRBV gene calls); cells with only one detected productive chain were also recorded. Contig- and clonotype-level outputs (filtered_contig_annotations.csv, clonotypes.csv) were joined to the scRNA-seq Seurat objects as metadata to link clonotypes to clusters and compute per-cluster and per-patient clonotype frequencies. In this manuscript, "productive TCR" refers to successful recombination and transcription (i.e., in-frame TRA/TRB V(D)J transcripts as above), whereas "surface TCR expression" refers to surface protein expression capable of antigen recognition (assembly of the CD3/TCR complex at the cell surface, distinct from transcript evidence).

Pseudobulk Differential Gene Expression Analysis

The cells of interest were subset from the full single-cell RNA-seq Seurat object. Cells were then grouped by sample into a pseudobulk matrix using Seurat's "AggregateExpression" function. Next, this matrix was used input into differential expression analysis using edgeR (v3.36.0) using the "glmLRT" function. Table "Fig. S1B" in our Supporting Data Values file shows the full results of this analysis. We visualized genes with $p<0.05$ significance in the reported volcano plot.

Differential Gene Expression Analysis with the Th2 Atlas

To identify differentially expressed genes between LHES and Th2 Atlas-derived cells, we used the FindMarkers function from the Seurat package (v4.3.0). Integration of the external Th2 Atlas cells with incomplete disease metadata precluded us from running pseudobulk analyses. Differential expression was therefore performed using the Wilcoxon rank-sum test. We visualized genes with $p<0.05$ significance in the reported volcano plot.

SCENIC

Single-cell regulatory network inference and clustering (SCENIC) identifies putative regulatory networks and provides a measure of "regulon activity" that can be high (suggesting a coordinated expression of TF target genes) or low (less coordinated expression). However, SCENIC itself does not provide direct experimental confirmation of upregulation or downregulation; it predicts regulatory interactions based on co-expression and motif analysis. To run this analysis on our dataset, cells of interest were subset from the full single-cell RNA-seq object. Following loom file conversion of the Seurat object subset, we executed the standard pySCENIC pipeline (v0.12.1) in three major steps: gene regulatory network inference, context-dependent enrichments using cis-regulatory motifs, and activity scoring of regulons in individual cells using the AUCell algorithm. We used the FindAllMarkers function, ensuring even sample representation with equal number of cells, to find differentially expressed regulons between responders and non-responders. We integrated the results of the subsequent SCENIC (8) analysis into our Seurat object. We report the complete results of this analysis, with all gene regulons and their respective p-values in each cluster, in tabs "Fig. 1E" and "Fig. S1C" of our Supportive Data Values file. For clarity and readability, we labeled biologically important regulons with statistically significant changes ($p < 0.05$) in heatmap visualizations.

CellChat

All cells from the tissue scRNA-seq dataset were made into a pseudobulk average expression Seurat object, grouped by sample and cell type. This was inputted into the CellChat(9) (1.4.0) pipeline using the human database accessed on April 29th, 2023. After running the standard CellChat workflow to estimate inter-cluster communication probabilities, the output visualised with CellChat's bubble-plot utility. Statistically significant interactions ($p <0.05$) between Clusters 1 and 2 were selected in the final visualization.

scRNA Variant Calling in CD3-CD4+ Cell Clusters

Cell barcodes corresponding to CD3-CD4+ transcriptional clusters were identified through unsupervised clustering of single-cell RNA sequencing data. Raw sequencing reads from these barcodes were aligned to the GRCh38 reference genome using STAR (v2.7.10a) with default parameters optimized for spliced reads. To facilitate accurate variant calling, BAM files were processed using GATK (v4.3.0.0) SplitNCigarReads to split reads at intron-exon junctions and hard-clip intronic sequences. Variants were subsequently called using GATK HaplotypeCaller. Resultant variant call format (VCF) files were annotated using ANNOVAR (2020Jun07) against RefSeq and COSMIC v97 to predict functional consequences and assess pathogenicity. Variants were manually reviewed using Integrative Genomics Viewer (IGV) to confirm expression specificity within the CD3-CD4+ clusters.

Integration of CD4 cells from LHES samples with Th2 Atlas

We performed systematic integration of CD4+ T-cells from LHES patients with the reference Th2 Atlas published by the Brennan Lab (10). Initial processing of the Th2 Atlas dataset began by replicating the established dimensionality reduction and Harmony batch correction methodology published. We then employed a reference-based integration approach, where the Th2 Atlas cells were the reference dataset and CD3-CD4+ T-cells served as the query dataset. Integration anchors were computed using the FindTransferAnchors function in Seurat v4, enabling robust identification of corresponding cell states between datasets. These anchors facilitated projection of query cells onto the reference space through the MapQuery function, allowing cluster identity prediction based on transcriptional similarity to established Th2 subsets. The integrated dataset underwent secondary preprocessing, including normalization, selection of 2,000 variable features, and principal component analysis. To optimize integration while preserving disease-specific variation, we applied a second round of Harmony batch correction ($\lambda = 1$, $\theta = 4$). Dimensionality reduction was performed using Uniform Manifold Approximation and Projection (UMAP) with 20 principal components, selected based on elbow plot analysis of principal component variance.

Amplicon Sequencing

Pre-treatment and post-treatment cells from Patient 3 were thawed with 8 mL of staining buffer (BioLegend, No. 420201). After centrifugation, cells were resuspended with 80 µL of staining buffer. Following the addition of 20 µL of FcX™ Fc blocking reagent, the cells were incubated for 10 minutes. After incubation, 6 µL of CD3 (BioLegend, No. 317314), CD4 (BioLegend, No.317415), CD8 (BioLegend, No. 301008) antibodies and incubated for 20 minutes at 4°C. $CD3^{-}/CD4^{+}/CD8^{-}$ gated-cells were sorted through FACSAria™ III Cell Sorter (BD). Sorted cells were lysed to get gDNA using QIAamp DNA Micro kit (Qiagen, No.56304). 100ng of gDNA was used as a template and PCR amplification of a 328 bp region was carried out to identify STAT3 DNA variant. The PCR primer was 5'-GGATGGATGCCCTGTTAGCA-3' and 5'-GTGTTTTGCGAGTCTGAGTGAA-3'. PCR products were extracted and purified through QIAEX II Gel Extraction Kit (Qiagen, No.20021). DNA was sequenced with the standard protocol using Plasmidsaurus Premium PCR Sequencing. Raw FASTQ reads were aligned to STAT3 and the

p.G618R point variant was identified. Reads quality scores > 30 were kept and the proportion of WT reads versus variants pre and post JAK inhibitor treatment were compared with the Chi-Square test.

siRNA-mediated Knockdown

To identify decreased CD3ζ expression affects down-regulation of CD3 expression, $CD3_{\zeta}$, $TCR_{\alpha/\beta}$, and NC siRNA was used. Jurkat cells (ATCC No. TIB-152) resuspended 2×10^5 in SE Cell Line 4D-Nucleofector buffer (Lonza, No. V4XC-1032) with siRNA targeting the human $CD3_{\zeta}$ or $TCR_{\alpha/\beta}$. The siRNA sequences were sourced from IDT and are as follows:

CD3ζ #1: 5'-CGCUUUCAGCGAAUGACAAAUCAT–3', 3'-GUGCGAAAGUCGCUUACUGUUUAGUA–5'

CD3ζ #2: 5'-GUCAUUCUCACUGCCUUGUUCCUGA-3', 3'-CACAGUAAGAGUGACGGAACAAGGACU-5'

CD3ζ #3: 5'-CCAGCUCUAUAACGAGCUCAAUCTA-3', 3'-UUGGUCGAGAUAUUGCUCGAGUUAGAU-5'

TCRα/β #1: 5'-GUGUCUGCUACCUUCUGGCACAACC-3', 3'-CGCACAGACGAUGGAAGACCGUGUUGG-5'

TCRα/β #2: 5'-GAACUGUUCCUUUACCGACUCUGCC-3', 3'-GACUUGACAAGGAAAUGGCUGAGACGG-5'

TCRα/β #3: 5'-GAGACCGACAUGAACCUGAACUUCC-3', 3'-AACUCUGGCUGUACUUGGACUUGAAGG-5'

Negative Control: IDT No. 51-01-14-03

3 kinds of siRNAs of each gene were combined at the same ratio and 300nM of combined siRNA was added. A 22 µl volume of this mixture was aliquoted to each well of a 96-well nucleofection plate (Lonza, No. V4XC-1032) and immediately electroporated using a 4D Lonza Nucleofector with program CL-120. Cells were resuspended in pre-warmed media and recovered for 15 min in the incubator before being transferred to culture. After 24 h incubation, cells were stained with Live/Dead™ Fixable Near-IR Dead cell stain kit (Invitrogen, No. L10119) for 15 min at 4°C. Wash cells twice and cells were stained with CD3ε (Biolegend, No. 317314) and TCRα/β (BIolegend, No. 109228) for 15 min at 4°C. Wash cells twice and cells were fixed by using IC fixation buffer (Invitrogen, No.00-8222-49) for 20 min at room temperature. Cells were washed twice and permeabilized using permeabilization buffer (Invitrogen, No. 00-8333-56). Cells were stained with $CD3_{\zeta}$ (Invitrogen, No.12-2479-80) for 30 min at room temperature. Cells were washed twice and measure $CD3_{\varepsilon}$, $CD3_{\zeta}$, $TCR_{\alpha/\beta}$ expression via flow cytometry.

Statistics Overview.

Statistical analyses were performed using R (version 4.4.0). Pseudobulk differential gene expression analysis of the RNA assay in LHES samples was conducted using the likelihood

ratio test from the edgeR package. Surface protein expression (ADT) and differential analysis comparing cells from LHES samples with the Th2 Atlas were performed using the Wilcoxon rank-sum test. Regulon activity was analyzed using the Wilcoxon rank-sum test on SCENIC AUC scores; the top 150 significant regulons per cluster were used for heatmap visualization. Cell–cell communication was inferred using CellChat, where the software determines statistical significance of interactions using a one-sided permutation test followed by Benjamini-Hochberg correction for multiple testing. Comparisons of variant frequencies in amplicon sequencing data were assessed using the chi-squared test. For the siRNA-mediated knockdown experiment, comparisons between conditions were analyzed using a 2-tailed Student's t-test. A P value < 0.05 was considered statistically significant for all tests unless otherwise specified.

Analysis	Samples Included	Details / Contrasts Performed	Relevant Analysis Parameters	Relevant Figures
CD4 clustering	P1_LHES, P2_LHES, P3_LHES_PRE, P3_LHES_POST, P4_LHES, P5_iHES.	Cells with detectable RNA expression were combined in one Seurat Object. CD4+ cells were subset.	The standard Seurat v4.3 Weighted Nearest Neighbor approach, integrating transcriptomic and protein expression modalities, was used.<br><br>The FindMultiModalNeighbors function was run on the Harmony-corrected principal components from RNA (cluster resolution = 0.3) and ADT (cluster resolution = 0.3) reductions.<br><br>The FindClusters function was used with the default SLM algorithm (algorithm = 3) and a resolution parameter of 0.5.	Figure 1B
ADT Heatmap	P1_LHES, P2_LHES, P3_LHES_PRE, P3_LHES_POST, P4_LHES	Naïve (C00), CD3-CD4+ (C01, C02), T regulatory (C03), and T memory (C04) clusters. All from CD4+ clusters.	The FindAllMarkers function was used with significantly upregulated genes (only.pos = TRUE) on the ADT assay.	Supplemental Figure 1A

RNA expression of CD3-CD4+ cells	P1_LHES, P2_LHES, P3_LHES_PRE, P3_LHES_POST, P4_LHES	Naïve (C00), CD3-CD4+ (C01, C02), T regulatory (C03) and T memory (C04) clusters. All from CD4+ clusters.	Expression data were unscaled (scale = FALSE).	Supplemental Figure 1D
Differential gene expression (edgeR pseudobulk)	P1_LHES, P2_LHES, P3_LHES_PRE, P4_LHES	CD3-CD4+ cells (C01 and C02 combined) vs CD3+CD4+ (defined as all other cells in CD4-gated Seurat object)	The RNA expression table inputted into edgeR were filtered to remove genes with fewer than 15 counts. We then performed standard pseudobulk analysis with the edgeR pipeline (DGEList, calcNormalFactors, estimateDisp, glmFIT, glmLRT functions) with default parameters.	Supplemental Figure 1B
Differential gene expression (Wilcoxon Rank-Sum Test)	P1_LHES, P2_LHES, P3_LHES_PRE, P4_LHES<br><br>Full Th2 Atlas	CD3-CD4+ cells (C01, C02) vs. Th2-memory-like cells from Th2 Atlas	FindMarkers function with default parameters.	Figure 1D
SCENIC	P1_LHES, P2_LHES, P3_LHES_PRE, P3_LHES_POST, P4_LHES	CD3-CD4+ cells (C01, C02) and Naïve CD4+ T-cells (C00)	The output of the standard pySCENIC pipeline from the SCENIC software was scaled for heatmap visualization. We ran FindAllMarkers with significantly upregulated genes (only.pos = TRUE).	Supplemental Figure 1C
	P1_LHES, P2_LHES, P3_LHES_PRE, P3_LHES_POST, P4_LHES<br><br>Full Th2 Atlas	CD3-CD4+ cells (C01, C02) vs Th2-memory-like cells from Th2 Atlas		Figure 1E
CellChat	P1_LHES, P2_LHES, P3_LHES_PRE, P3_LHES_POST, P4_LHES	C01 CD3-CD4+ cells vs C02 CD3-CD4+ cells	Gene-expression filter > 10% of cells, computeCommunProb was run on the normalized expression matrix.	Figure 1F

			Otherwise default parameters were used.	
scRNA DNA variant calling of CD3-CD4+	P3_LHES_PRE, P3_LHES_POST	DNA variant calling was performed for each sample individually. For each sample, we compared variants found in cells from the CD3-CD4+ clusters to those of non CD3-CD4+ clusters as a control.	Default parameters were used with the STAR alignment and GATK SplitNCigarReads and HaplotypeCaller softwares.	Figure 1H

Study Approval. All patient samples were collected under protocols approved by the local Institutional Review Boards from participating institutions in accordance with the ethical principles put forth in the Declaration of Helsinki. All relevant ethical regulations for work with human participants were complied with and informed consent was obtained. This study was reviewed and approved in compliance with the Northwestern University Institutional Review Board. Research participants provided informed consent for publication of images and the records of informed consent have been retained. Participating institutions included Northwestern Memorial Hospital.

Data Availability. The single-cell sequencing data have been deposited in the database of Genotypes and Phenotypes (dbGaP) under the accession code phs004041.v1.p1 and are available. All the other data supporting the findings of this study are available within the article and supplementary files or from the corresponding author upon reasonable request. Source data for the figures in this article are available as a Supporting Data Values File.

References

1. Shomali W, and Gotlib J. World Health Organization and International Consensus Classification of eosinophilic disorders: 2024 update on diagnosis, risk stratification, and management. *Am J Hematol.* 2024;99(5):946-68.

2. King B, Lee AI, and Choi J. Treatment of Hypereosinophilic Syndrome with Cutaneous Involvement with the JAK Inhibitors Tofacitinib and Ruxolitinib. *Journal of Investigative Dermatology.* 2017;137(4):951-4.
3. Walker S, Wang C, Walradt T, Hong BS, Tanner JR, Levinsohn JL, et al. Identification of a gain-of-function STAT3 mutation (p.Y640F) in lymphocytic variant hypereosinophilic syndrome. *Blood.* 2016;127(7):948-51.
4. Reinstein ZZ, Zhang Y, Ospina OE, Nichols MD, Chu VA, Pulido AM, et al. Preexisting Skin-Resident CD8 and gammadelta T-cell Circuits Mediate Immune Response in Merkel Cell Carcinoma and Predict Immunotherapy Efficacy. *Cancer Discov.* 2024;14(9):1631-52.
5. Fleming SJ, Chaffin MD, Arduini A, Akkad AD, Banks E, Marioni JC, et al. Unsupervised removal of systematic background noise from droplet-based single-cell experiments using CellBender. *Nat Methods.* 2023;20(9):1323-35.
6. McGinnis CS, Murrow LM, and Gartner ZJ. DoubletFinder: Doublet Detection in Single-Cell RNA Sequencing Data Using Artificial Nearest Neighbors. *Cell Syst.* 2019;8(4):329-37 e4.
7. Andreatta M, Corria-Osorio J, Muller S, Cubas R, Coukos G, and Carmona SJ. Interpretation of T cell states from single-cell transcriptomics data using reference atlases. *Nat Commun.* 2021;12(1):2965.
8. Aibar S, Gonzalez-Blas CB, Moerman T, Huynh-Thu VA, Imrichova H, Hulselmans G, et al. SCENIC: single-cell regulatory network inference and clustering. *Nat Methods.* 2017;14(11):1083-6.
9. Jin S, Guerrero-Juarez CF, Zhang L, Chang I, Ramos R, Kuan C-H, et al. Inference and analysis of cell-cell communication using CellChat. *Nature Communications.* 2021;12(1):1088.
10. Kratchmarov R, Djeddi S, Dunlap G, He W, Jia X, Burk CM, et al. TCF1-LEF1 co-expression identifies a multipotent progenitor cell (T(H)2-MPP) across human allergic diseases. *Nat Immunol.* 2024;25(5):902-15.
